# The Effect of Dexmedetomidine in Combination with Bupivacaine on Sensory and Motor Block Time and Pain Score in Supraclavicular Block

**DOI:** 10.1155/2021/8858312

**Published:** 2021-04-10

**Authors:** Shahryar Sane, Shahram Shokouhi, Parang Golabi, Mona Rezaeian, Behzad Kazemi Haki

**Affiliations:** ^1^Department of Anesthesiology, Urmia Imam Khomeini Hospital, Urmia University of Medical Sciences, Urmia, Iran; ^2^Department of Anesthesiology, Mahabad Imam Khomeini Hospital, Urmia University of Medical Sciences, Mahabad, Iran

## Abstract

**Background:**

Brachial plexus block is frequently recommended for upper limb surgeries. Many drugs have been used as adjuvants to prolong the duration of the block. This study aimed to assess the effect of dexmedetomidine with bupivacaine combination and only bupivacaine on sensory and motor block duration time, pain score, and hemodynamic variations in the supraclavicular block in upper extremity orthopedic surgery.

**Methods:**

This prospective, double-blind clinical trial study was conducted on 60 patients, 20 to 60 years old. Patients were candidates for upper extremity orthopedic surgeries. The sensory and motor block were evaluated by using the pinprick method and the modified Bromage scale. The postoperative pain was assessed by utilizing a visual analog scale.

**Results:**

The mean onset time of sensory and motor block in patients receiving only bupivacaine was, respectively, 31.03 ± 9.65 min and 24.66 ± 9.2 min, and in the dexmedetomidine receiving group, it was about 21.36 ± 8.34 min and 15.93 ± 6.36 minutes. The changes in heart rate and mean arterial blood pressure were similar in both groups. The duration of sensory and motor block and the time of the first analgesia request in the intervention group were longer. Postoperative pain was lower in the intervention group for 24 hours (*P* = 0.001).

**Conclusion:**

Dexmedetomidine plus bupivacaine reduced the onset time of sense and motor blocks and increased numbness and immobility duration. Also, dexmedetomidine reduced postoperative pain significantly with the use of bupivacaine for supraclavicular blocks. *Trial Registration*. IRCT, IRCT20160430027677N15. Registered 05/28/2019, https://www.irct.ir/trial/39463.

## 1. Background

The supraclavicular block is used extensively and effectively for the distal upper extremity surgical operation [1, 2]. This technique is used with general anesthesia or alone as an anesthesia method for distal limb surgery with very low complication. However, regarding the effectiveness of this method in upper extremity surgery, various reports have been presented. But so far, selective drug combination for the supraclavicular block has not been considered [1]. To increase the duration of local anesthetic effect in the supraclavicular block, epinephrine, *α*2 agonist, corticosteroids, bicarbonate, and opioids have been used. In the meantime, epinephrine is mostly used. Although epinephrine reduces the absorption of local anesthetics, reduces their toxicity, and prolongs anesthetic duration, it can cause hypertension and tachycardia. Therefore, its usage is limited when the patients have cardiovascular disease or hyperthyroidism [3–6]. The rare complications of supraclavicular nerve block are pneumothorax, phrenic nerve block, Horner syndrome, neuropathy, and nerve damage [7, 8].

2Using additive drugs in local anesthetic can reduce the dosage of local anesthetic drugs for nerve block and reduce the probable side effects of these drugs, and increasing the benefits of adjuvant drugs can be used. The new drugs have been used in this field, including buprenorphine [9], dexamethasone [10], magnesium [11], and midazolam [12]. The use of these drugs to reduce onset block time, increasing the duration time of analgesia without incidence of unwanted systemic complications, motor block prolongation, and finally reducing the total dose of local anesthetics drugs, has been proposed and studied. Recently, alpha-2 receptor-stimulating drugs due to excellent sedative effects, analgesia, and anesthesia with hemodynamic stability have been considered [13].

Bupivacaine is a potent local anesthetic with unique characteristics from the amide group of local anesthetics, which was first discovered in 1957 and widely used for prolonged local and regional anesthesia. [14].

The dexmedetomidine is an active D-isomer of medetomidine and is similarly related to clonidine. Dexmedetomidine is a specific Alpha2 agonist with an *α*2 : *α*1 ratio of 1620 : 1 and metabolized in two ways via liver glucuronidation and cytochrome P450 [15–17]. Dexmedetomidine was recently added to the drugs that have been used in the nerve block, such as bupivacaine. Singh et al. in 2016 investigated the effect of combining dexmedetomidine with levobupivacaine [18]. Tripathi et al. in 2016 compared the effect of adding dexmedetomidine and clonidine to bupivacaine in brachial plexus block for upper extremity surgery [19]. They concluded that using dexmedetomidine reduces the onset time of the sensory and motor block. And it also prolongs the duration time of anesthesia and analgesia and increases the quality of anesthesia in the block.

Some studies have been conducted on the use of local anesthetics with additive agents [3, 9–12, 20]. However, a unit drug for adding local anesthetic to improve block quality is not recommended [3, 20, 21]. Two meta-analyses of randomized controlled trials compared 25 trials about the efficacy and safety of dexmedetomidine. Finally, the study concludes that further research should focus on the effectiveness and safety of the preneural administration of dexmedetomidine [3, 20]. According to the mentioned items, this study assessed the effect of dexmedetomidine combination with bupivacaine and only bupivacaine on sensory and motor block duration time, pain score, and hemodynamic variations supraclavicular block in upper extremity orthopedic surgery. Our study's primary endpoints were sensory and motor block quality, as assessed by the pinprick method and Bromage scale. And postoperative pain was evaluated by the visual analog scale (VAS) [22]. The secondary outcomes included the effect of dexmedetomidine on hemodynamics, complications, and first analgesic request time.

## 2. Methods

### 2.1. Study Design

This randomized, double-blind prospective clinical trial study was approved by the Research and Ethics Committee of the Urmia University of Medical Sciences (IR.UMSU.REC.1397.181) and registered in the Iranian Registry of Clinical Trials (IRCTID: IRCT20160430027677N15). This study was conducted on 60 patients 20 to 60 years old with ASA classes I and II. Patients who were candidates for upper extremity orthopedic surgeries under the supraclavicular block were divided into two groups (intervention and control groups). According to the random number table, the anesthesiologist did not know which patient belonged to which group.

### 2.2. Subjects and Setting

All patients were visited by an anesthesiologist the day before the surgery. All patients were adequately explained and educated about the visual analog scale (VAS) (zero: no pain and ten: the worst pain ever experienced). In this study, patients were excluded from the study with the following criteria: the history of central nervous system disease or neuromuscular disorders; psychiatric; renal dysfunction; respiratory disease; cardiovascular disease; hepatic disease; pregnant women, lactating mothers; a history of allergy to any drugs, diabetics; patients who were contraindicated for performing a supraclavicular block such as coagulopathy; local infection; and patients' dissatisfaction of technique.

#### 2.2.1. Intervention Design

Intervention group: patients in the dexmedetomidine group (intervention group) received 39 ml of bupivacaine (0.25%) + 0.75 *μ*g/kg dexmedetomidine (total volume 40 ml).

Control group: patients in the control group, received 39 ml of 0.25% bupivacaine + 1 ml normal saline (total volume 40 ml).

Patients were kept fasting for at least 8 hours before surgery. The patients, surgeons, and anesthesiologists were blinded to the division of the groups.

A standard pulse oximetry monitor, a noninvasive blood pressure measurement system, and an electrocardiogram were attached to patients in the operating room. Baseline heart rate, blood pressure, and oxygen saturation were recorded. After inserting an 18 cm venous catheter on the nonoperative arm before performing supraclavicular block, all patients premedicated with midazolam 0.04 mg/kg and received oxygen 5 L/min. They were placed in the supine position and slightly turned 45° their heads to the contralateral side. The upper arm was abducted at 90°. All aseptic precautions were conducted before performing the procedure. The supraclavicular plexus site was determined using a nerve stimulator (Stimuplex; B. Braun Melsungen, Melsungen, Germany) attached to a 22-gauge, 55 mm long stimulating needle (Stimuplex D; B. Braun Melsungen, Melsungen, Germany) under ultrasonic apparatus. The needle's location was considered acceptable when the output current of 0.5 mA still produced a suitable motor response in the distal of the limb.

The solution was injected under the guidance of the ultrasonic apparatus. Sensory and motor blocks were assessed every 3 min to the first 30 minutes after full drug injection. The surgery was allowed when the block was determined to be sufficient.

All vital parameters, including HR, NIBP, RR, and SPO2, were recorded in the checklist every 5 min to the first 30 min and then every 10 min to the end of surgery.

### 2.3. Variables

Sensory and motor block and vital signs were measured instantly after surgery in the recovery room (where the block was done). Sensory blockade of each nerve evaluated by pinprick method in sensory dermatomes related to the sensory areas and graded as score 0 = no sensation score 1 = dull sensation score 2 = sharp pain felt [22]. Motor blockade was evaluated by using a modified Bromage scale as 3 = elbow flexion against gravity force, 2 = wrist flexion against gravity force, 1 = finger movement, and 0 = no motion [22]. The onset of sensory block was defined as the time between the local anesthetic administration till dull sensation to pinprick test. The onset time of motor block was considered as the time between injection till Bromage score 2. The sensory block's duration time was determined as the sensory block's time interval onset until the first pain sensation in the Pinprick test. The motor block's duration time was described as the time interval between the complete motor paralysis and the limb's full movement. Patients' postoperative pain perception was assessed by the visual analog scale (VAS), which was explained to the patients by scored pain severity between zero and 10 (zero, no pain, and 10, worst pain imaginable). The severity of pain was measured and recorded in the recovery at 6, 12, and 24 hours after surgery. When the VAS score was higher than 4, analgesia was administered. The time of local anesthetic injection and the first analgesic administration was considered the duration of analgesia. Patients were monitored for side effects such as hypotension (a 20% decrease from baseline value), bradycardia (HR < 50), hypoxemia (SpO2 < 90%), nausea, and vomiting and were recorded in the questionnaire.

### 2.4. Statistical Analysis

Based on the Tripathi et al. study in 2016 [19] and considering the power (probability) test 80% and confidence interval 95% (*α* = 0.05 *β*10% = ), the sample size was determined of 30 patients in each group. To provide descriptive features, tables, frequency charts, and descriptive statistics, including mean and standard deviation, were used. For normal data, the Repeated Measures test was used to compare the mean pain at 6, 12, and 24 hours after surgery. Furthermore, the Friedman test was used for nonnormal data. In this study, to investigate qualitative variables such as gender, the Chi-square test was used. Moreover, for quantitative variables in two groups, an independent *t*-test was used for normal data. For nonnormal data, the Mann–Whitney test was used. The normality of data was tested using the Kolmogorov–Smirnov test. The results were analyzed by SPSS software version 23, and a *P* value ≤ of 0.05 was considered significant.

## 3. Result

The patient's demographics data in the two groups are presented in Table 1. According to Table 1 data and the chi-square test, no statistically significant difference between the two groups characterizes data including gender, weight, age, and kind of surgery (*P* > 0.05).

### 3.1. Sensory and Motor Block Onset Time

The onset time of the upper extremity sensory block in the intervention group was shorter than the control group, and this difference was significant (*P* = 0.026). Also, the onset time in the upper extremity motor block in the control group was longer than the intervention group, and it was statistically significant (*P* = 0.041).

### 3.2. Sensory and Motor Block Score

According to the nonnormal distribution of data in the Mann–Whitney test, the mean score of the sensory block in the control group was 31.25 with a total of 937.5 and in the intervention group, the mean score and the total score were respectively 29.75 and 892.5. This difference was not significant (*P* = 0.809), but the score was less in the intervention group (Table 2).

The mean score of the motor block (Bromage score) in the control group was 35.58 with a total score of 10.567, and in the intervention group, the mean score was 25.42 with a total score of 765.5. This difference was statistically significant (*P* = 0.012), and the mean score was lower in the intervention group (Table 2).

### 3.3. Sensory and Motor Block Duration Time

In the bupivacaine group, the duration time of sensory block in the upper extremity was 333.5 ± 94.35 min, and in the intervention group, it was 475 ± 137.5 minutes. In the intervention group, the duration time of anesthesia was longer than the control group, and this difference was statistically significant (*P* = 0.022).

In the control group, the duration time of motor block was 317 ± 10.52 minutes, and in the intervention group, it was 488 ± 157.5 minutes, and this difference was significant (*P* = 0.03).

**Figure 1:**
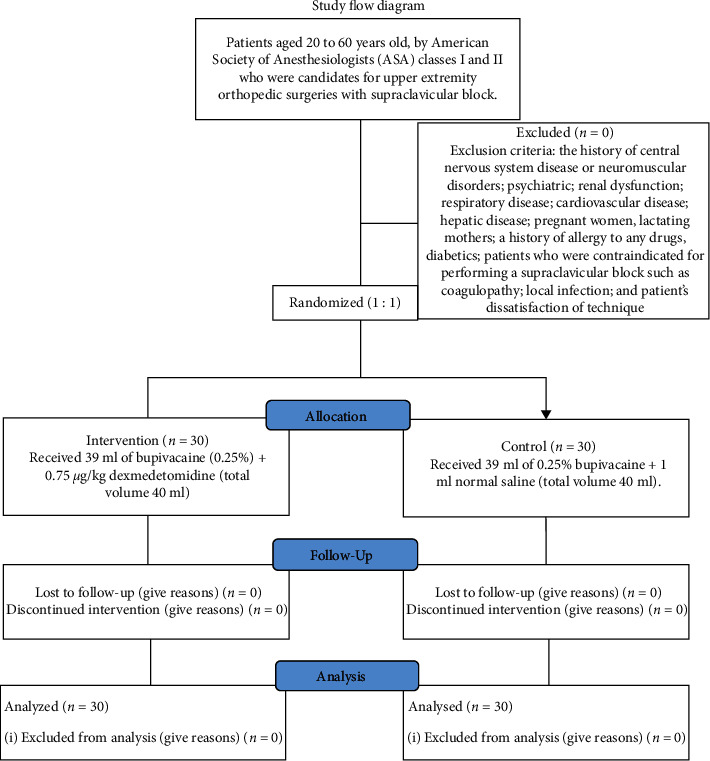
Study flow diagram.

The first analgesia request in the control group was 308 ± 109.14 minutes, and in the intervention group, it was 458 ± 205/43 minutes. The first analgesic request in the intervention group was more than the control group, and this difference was significant (*P* = 0.001) (Figure 1).

### 3.4. Heart Rate Variation

**Figure 2:**
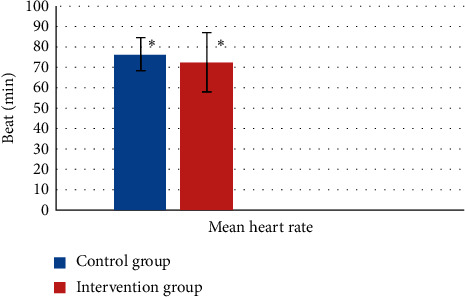
The mean of heart rate variations in both groups during the study period. ^∗^*P* = 0.454 and not significant.

The mean of heart rate variations at the outset and the end of the surgical procedure was reduced in both groups: in the intervention group, it was 72.45 ± 8.05 beats and in the control group, it was 76.3 ± 14.4 beats. In the conducted independent *t*-test, it was not significant (*P* = 0.454) (Figure 2).

### 3.5. Mean Arterial Pressure Changes

**Figure 3:**
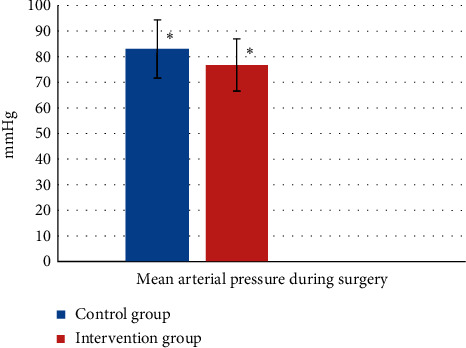
Mean arterial pressure during surgery in both groups during the study period. ^∗^*P* = 0.123 and not significant.

The mean arterial pressure changes during surgery in the intervention group were 83.24 ± 11.36 mm Hg. The control group was 76.93 ± 10.06 mm Hg, which in conducted independent *t*-test, it was not significant (*P* = 0.123) (Figure 3).

### 3.6. Pain Score after Surgery

**Figure 4:**
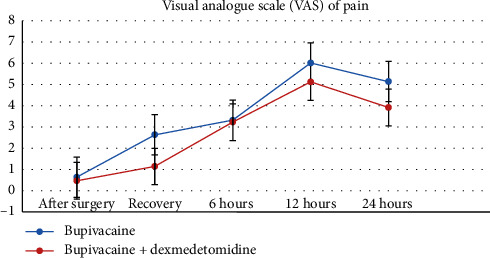
Comparison of visual analog scale (VAS) of pain in patients' groups within 24 hours after surgery. The VAS score in all hours of study in the intervention group was lesser than the control group and it was significant *P* = 0.001.

Mean pain score based on VAS after surgery; at recovery; and 6 hours, 12 hours, and 24 hours after surgery in the control group (receiving bupivacaine alone) were 0.633, 2.633, 3.313, 6.017, and 5.11, respectively and in the intervention group (bupivacaine + dexmedetomidine), they were 0.47, 1.14, 3.23, 5.12, and 3.92, respectively (Figure 4). In the Two-Way Repeated Measure ANOVA test with *P* = 0.001, this difference in inpatient pain evaluation was significant. In all hours of study in the intervention group, it was lesser than the control group.

### 3.7. Complications

**Figure 5:**
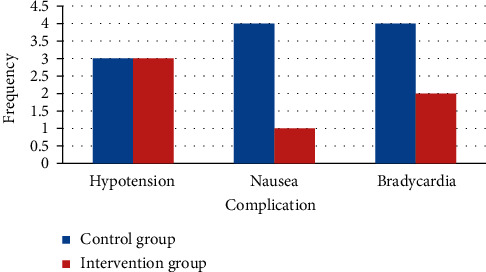
Frequency of complications in two groups. The difference in all of the side effects frequency was not significant (*P* > 0.05).

Hypotension occurred in 3 patients in both intervention and control groups that were not statically significant (*P* = 0.217). Nausea in 4 patients in the control group and one patient in the intervention group was observed, which was not significant (*P* = 0.353). 4 patients in the control group and two patients in the intervention group had bradycardia. It was not significant (*P* = 0.554) (Figure 5).

## 4. Discussion

Rapid onset time and prolonged analgesia and motor block without adverse effects highlighted an ideal local block. Hence, many various drugs have been added to topical anesthetic drugs as an adjuvant. Clonidine has been used as an *α*2 agonist with ropivacaine in the axillary block [23]. In recent studies, the administration of dexmedetomidine has been described as an effective drug in increasing the block's time [24, 25]. It also increased the risk of bradycardia, hypotension, and drowsiness [3].

The activation of the presynaptic receptor in the central nervous system prevents the norepinephrine release and pain signals [26]. Dexmedetomidine produces a natural sleep for the patient with an effect on the Locus coeruleus [27, 28]. Dexmedetomidine has been used without nerve damage. In the Brummett et al. study done on rats, after 24 hours and 14 months, axons and myelin have been reported as no damage [29].

In the Gandhi et al. study in 2012 conducted on 70 patients, the sensory block's onset time was shorter in the bupivacaine group than bupivacaine with dexmedetomidine. Also, in the control group, the motor block's onset time was faster than the dexmedetomidine group. In the dexmedetomidine group, the duration time of sensory and motor block was prolonged. Mean arterial pressure and heart rate variations were similar between the two groups, and the duration time of analgesia in the control group was shorter than the intervention group (dexmedetomidine) [30]. The results of this study are consistent with our findings.

In this study, the reason for the earlier onset of the motor block than a sensory block is motor fibers' presence in the nerves' outer layers in front of the central sensory fibers. Winnie and Nader explained the study [31]. In the study conducted on 50 patients in 2014 by Agarwal et al., the onset time of sensory block and limb immobilization in the group who received dexmedetomidine with bupivacaine was shorter than only bupivacaine, and also the duration time of sensory and motor block in the group who received dexmedetomidine was longer. The duration time of analgesia in the dexmedetomidine group was prolonged compared to the control group. This study's results are consistent with our findings; however, the dose of the used drug in the two studies is different [32].

Bharti et al. conducted a study on 60 patients in two groups: control group (ropivacaine and lidocaine with adrenaline) and intervention groups (dexmedetomidine 1 *μ*g/kg plus other drugs); the onset time of sensory block was similar in the two groups. The motor block's onset time was shorter in the receiving dexmedetomidine group. The sensory and motor block duration was longer in the dexmedetomidine group, which had reduced postoperative pain and reduced the need for analgesia in the intervention group [33]. In the present study, the sensory block's onset time was shorter in the dexmedetomidine group, and this difference was statistically significant. In other measured parameters, the results of the two studies were not different. It seems no difference in the onset time of sensory block in the two groups due to using the low dose of dexmedetomidine combined with the high volume of other drugs that have reduced the effective dose of local dexmedetomidine.

In a recent meta-analysis conducted by Abdallah and Brull, adding dexmedetomidine to other drugs has been reported to prolong the brachial plexus' motor block and prolonged the postoperative analgesia [34]. The results of this study are consistent with our study findings.

In separate studies conducted by Kathuria et al., dexmedetomidine with ropivacaine had improved the onset time of sensory and motor block. It increased the duration time sensory block and motor block compared with ropivacaine [35].

In our study, dexmedetomidine reduced sensory and motor blocks' onset time and increased sensory and motor blocks' duration time. This finding was similar to previous studies. The decrease of the onset time of sensory and motor block in the present study and the inconsistent results of previous studies due to the use of multiple drugs at the same time and the difference in the definition of the onset time of sensory and motor block, however, in Gandhi's study did not provide a precise definition of the onset time of sensory and motor block [30].

In explaining dexmedetomidine's mechanism in previous studies on rats, cationic hyperpolarization blocks and maintenance of nerve stimulation have been attributed to prolonged sensory and motor blocks [29]. In a study performed by Kosugi et al. on *α*2 agonist, the intravenous dose required for nerve block has reported more than 1000 times of topical dose, and they have reported the effect of dexmedetomidine with local anesthetic is through vasoconstriction, delayed in local anesthetic uptake as well as nerve conduction direct block [36].

In another study conducted by Fritsch et al. in 2014, they have reported the use of dexmedetomidine with ropivacaine in interscalene block, decreasing postoperative pain and prolonged block time [37]. Also, in our study, postoperative pain reduction was more prominent in the dexmedetomidine group.

Postoperative pain score in all hours of the present study in the intervention group was lesser than the control group. In Bharti et al.'s study, pain score was comparable among groups except at 8 and 10 hours, and pain scores were lower in the dexmedetomidine group versus the control group [33]. The results of this study are consistent with our study findings. In Lee et al.'s study that used the MgSO4 with Bupivacaine, they illustrated no differences in VAS scores between the two groups [11].

Hypotension and bradycardia are the most common side effect observed with *α*2 agonists. In a study that Esmaglu and his colleagues had done, adding 100 *μ*g of dexmedetomidine to levobupivacaine had caused bradycardia in 7 of the 30 patients [38]. In Kwon's study, heart rate and mean arterial pressure in the dexmedetomidine group had decreased significantly [39] whereas in our study, this decrease occurred in mean arterial pressure and mean heart rate, and it was not statistically significant.

In our study, bradycardia was observed in 4 of the 30 patients in the intervention group, which seems to be due to the low dose of dexmedetomidine. Hypotension in 3 patients has occurred in both groups, and this difference was not statistically significant. However, in previously conducted studies, the use of dexmedetomidine was not associated with hypotension and bradycardia [40, 41].

## 5. Conclusion

Dexmedetomidine with bupivacaine in the supraclavicular block effectively reduced the onset time of sensory and motor blocks. It increased sensory and motor blocks' duration time without considerable side effects such as hypotension and bradycardia. Besides, dexmedetomidine significantly reduced postoperative pain in the dexmedetomidine with the bupivacaine group.

### 5.1. Limitations of Our Study Included

Our study's limitations were the no measurement of dexmedetomidine serum dose during surgery that would make the evaluation of this drug's systemic effect unpredictable after local absorption and evaluation of another group of patients with receiving intravenous dexmedetomidine in future studies will resolve this restriction.

Increased surgery duration and general anesthesia needs were other limitations of the present study, leading to the exclusion of these cases.

## Figures and Tables

**Table 1:** Studied patients' demographic data and surgical characteristics.

	Intervention group *N* = 30 patients		Control group *N* = 30 patients		*P* value
Gender (F/M)	Female	Male	Female	Male	0.133
	12	18	7	23	
Age (year)	39.5 ± 14.9		34.7 ± 10.8		0.165

Kind of surgery	Soft tissue	Bone tissue	Soft tissue	Bone tissue	0.398
	14	16	16	14	
Weight (kg)	76.72 ± 18.9		73.75 ± 24.9		0.9

Values are mean ± SD or number of patients and kind of surgery. There are no significant differences between the two groups. Intervention group = dexmedetomidine + bupivacaine and control group = bupivacaine alone.

**Table 2:** Patients' supraclavicular block characteristics in two groups.

	Control group	Intervention group	*P* value
Onset time of motor block	24.66 ± 9.2 min	15.93 ± 6.36 min	*P* = 0.041
Onset time of sensory block	31.03 ± 9.65 min	21.36 ± 8.34 min	*P* = 0.026
Duration time of motor block	317 ± 10.52 min	488 ± 157.5 min	*P* = 0.03
Duration time of sensory block	333.5 ± 94.35 min	475 ± 137.5 min	*P* = 0.022
First analgesia request	308 ± 109. 14 min	458 ± 205/43 min	*P* = 0.001
Score of sensory block	31.25	29.75	*P* = 0.809
Score of motor block	35.58	25.42	*P* = 0.012

Values are mean ± SD or mean of sensory and motor block score. Intervention group = dexmedetomidine + bupivacaine and control group = bupivacaine alone. *P* value ≤0.05 is significant.

## Data Availability

All relevant data are included in the article. Additional information is available from the corresponding author on reasonable request.
